# Coronary artery lesions in pediatric scrub typhus: A case series from Guangzhou, China

**DOI:** 10.1371/journal.pntd.0014784

**Published:** 2026-10-07

**Authors:** Shiying Liu, Qing Wang, Yu Gong, Xufang Li, Fansen Zeng, Yi Xu

**Affiliations:** Department of Infectious Disease, Guangzhou Women and Children’s Medical Center, Guangzhou Medical University, Guangzhou, China; Yale University, UNITED STATES OF AMERICA

## Abstract

**Background:**

Scrub typhus, caused by Orientia tsutsugamushi, is a mite-borne rickettsial infection endemic to the Asia-Pacific region. While its systemic complications are well documented, coronary artery lesions (CALs) associated with scrub typhus in children remain poorly characterized. This study aimed to describe the clinical features, diagnostic challenges, and treatment outcomes of pediatric scrub typhus complicated by CALs.

**Methodology/Principal Findings:**

We retrospectively analyzed seven pediatric patients (aged 11 months to 8 years) with scrub typhus and echocardiographically confirmed CALs, admitted to Guangzhou Women and Children’s Medical Center, Guangzhou Medical University between 1January 2020 and 30 May 2026. Between 15 June 2025 and 1 July 2026, clinical data including epidemiological history, laboratory findings, imaging results, treatment regimens, and outcomes were collected from electronic medical records.

**Conclusions/Significance:**

Among the seven cases, CAL manifestations included coronary artery aneurysm (n = 2), coronary artery dilatation (n = 2), and coronary artery intimal thickening (n = 3). All seven patients were initially suspected of having Kawasaki disease.Six patients received intravenous immunoglobulin (IVIG), but all failed to respond. Among these six, four met the diagnostic criteria for Kawasaki disease (KD) or incomplete KD. Ultimately, all seven patients—including the one who did not receive IVIG—defervesced within 1–3 days of initiating antimicrobial therapy directed at scrub typhus.At follow-up, five patients with available echocardiography showed normalization of coronary arteries within one week to three months. These findings suggest that O. tsutsugamushi infection can trigger CALs indistinguishable from KD and that pathogen-directed therapy, rather than IVIG alone, is critical for fever resolution in such cases. Clinicians should maintain a high index of suspicion for scrub typhus in pediatric patients presenting with CALs, particularly when IVIG response is suboptimal.

## Author summary

Scrub typhus is a neglected tropical disease transmitted by infected mite larvae (chiggers) and is widespread across China and the Asia-Pacific region. Although it typically causes fever, rash, and multi-organ dysfunction, its capacity to damage coronary arteries in children — a complication most commonly associated with Kawasaki disease (KD) — has rarely been reported. In this study, we analyzed seven children treated at a major pediatric center in Guangzhou, China, who developed coronary artery aneurysms, dilatation, or intimal thickening following Orientia tsutsugamushi infection. Four patients who met the diagnostic criteria for KD or incomplete KD continued to have high fevers after standard immunoglobulin treatment, only improving once antibiotics targeting the bacterium were started. All patients eventually recovered. Among the six patients who underwent follow-up imaging, five showed normalization of coronary artery abnormalities. Our findings highlight that scrub typhus should be considered as a potential cause of coronary artery lesions in children, especially in endemic regions, and emphasize the importance of early microbiological diagnosis and specific antimicrobial therapy.

## Introduction

Scrub typhus (tsutsugamushi disease) is an acute febrile illness caused by the obligate intracellular bacterium Orientia tsutsugamushi and transmitted through the bite of infected larval trombiculid mites (chiggers) [1]. The disease is endemic across the Asia-Pacific region, extending from Japan and Korea to northern Australia and from the Indian subcontinent to the Pacific islands, collectively affecting an estimated one billion people at risk and causing approximately one million cases annually [1]. The infection triggers a systemic vasculitis with perivascular inflammation, endothelial dysfunction, and vascular leakage that can progress to multi-organ failure. Approximately one-third of patients develop organ dysfunction, including respiratory failure, shock, renal or hepatic impairment, central nervous system involvement, and hematologic abnormalities. Although mild cases generally carry a favorable prognosis, mortality among severe cases with multi-organ failure can reach 24% [2], and historical data from China have reported an overall mortality rate of 13.8% in untreated patients [3].

Kawasaki disease (KD), an acute vasculitis predominantly affecting children under five years of age, is characterized by fever, rash, conjunctivitis, mucositis, cervical lymphadenopathy, and extremity changes. Approximately 20% of untreated KD patients develop coronary artery aneurysms (CAA), making KD the leading cause of acquired heart disease in children in developed countries [4]. Although the etiology of KD remains incompletely understood, accumulating evidence implicates infectious triggers that provoke a dysregulated immune response in genetically susceptible individuals [5].

The overlapping clinical features of scrub typhus and KD — including prolonged fever, rash, lymphadenopathy, and coronary artery involvement — create significant diagnostic challenges, particularly in endemic regions. Scrub typhus misdiagnosed as KD has been documented [6], and concurrent scrub typhus and KD has been recognized as a rare but clinically important phenomenon [7]. However, systematic characterization of CALs attributable to O. tsutsugamushi infection in children remains limited. Here, we report a case series of seven pediatric patients with scrub typhus complicated by CALs treated at a tertiary children’s hospital in Guangzhou, China, and discuss the diagnostic and therapeutic implications of this underrecognized association.

## Methods

### Ethics statement

This study was approved by the Institutional Ethics Committee of Guangzhou Women and Children’s Medical Center, Guangzhou Medical University. All participants were recruited from our institution, which is a teaching hospital. Written informed consent was obtained from the parents or legal guardians of all minor participants during their hospitalization at our teaching hospital. All consent forms were signed after January 1, 2020. Authors had access to information that could identify individual participants during or after data collection.

### Study design and setting

We retrospectively reviewed the medical records of pediatric patients diagnosed with scrub typhus and concurrent CALs at Guangzhou Women and Children’s Medical Center, Guangzhou Medical University, between January 1,2020 and May 30,2026. Clinical data including epidemiological history, laboratory findings, imaging results, treatment regimens, and outcomes were collected from electronic medical records. The data collection period was 15 June 2025 to 1 July 2026.This study was approved by the Institutional Ethics Committee of Guangzhou Women and Children’s Medical Center, Guangzhou Medical University.

### Inclusion criteria

Eligible patients met both of the following criteria: (i) a confirmed or clinical diagnosis of scrub typhus according to the 2024 Chinese Expert Consensus on Clinical Diagnosis and Treatment of Scrub Typhus [8], based on epidemiological exposure history (travel to endemic areas, outdoor activities, grassland or bush contact, or rodent exposure within 3 weeks preceding illness onset), characteristic clinical manifestations (high fever, lymphadenopathy, rash, and pathognomonic eschar or ulcer), and laboratory confirmation (single serum Weil-Felix test OXK titer ≥ 1:160, PCR detection, or pathogen isolation); and (ii) echocardiographic evidence of CALs as defined by the 2017 American Heart Association criteria [9] or the presence of coronary artery intimal thickening with perivascular echogenicity [10]. Specifically, CAA was defined as a Z-score ≥ 2.5; coronary artery dilatation as a Z-score between 2.0 and 2.5; and coronary artery intimal thickening as exceeding the age-adjusted upper limits of normal (days 31-365: 23% [maximum 57%]; 1-15 years: 20% [maximum 38%]) [10].

### Exclusion criteria

Patients were excluded if they had (i) lymphadenopathy, anemia, thrombocytopenia, or neutropenia attributable to other etiologies; (ii) known immunodeficiency disorders; or (iii) incomplete clinical records.

### Data collection

Clinical data — including demographics, epidemiological history, symptoms and signs, laboratory investigations, echocardiographic findings, treatment regimens, and outcomes — were extracted from the hospital electronic medical record system, clinical report database, and imaging archiving system.

### Statistical analysis

Given the small sample size, data were analyzed descriptively. Continuous variables are presented as ranges, and categorical variables as counts.

## Results

### Demographic and epidemiological characteristics

The study cohort comprised seven patients (two males, five females) with a median age of 3.0 years (range: 11 months to 8 years). Illness onset occurred in January (n = 1), May (n = 1), June (n = 4), and September (n = 1). All patients were long-term residents of Guangdong Province, China.

Six of seven patients reported potential epidemiological exposures: two had a history of mosquito bites and four reported outdoor activities involving grass or vegetable fields.

### Clinical manifestations

The median duration from symptom onset to hospital admission was 9 days (range: 7-14 days). All seven patients presented with fever (peak temperature range: 39.1-42.0 degrees C); fever was the initial symptom in six patients, while one presented with cough. All patients had rash, including six with the characteristic eschar (Fig 1). Bilateral cervical lymphadenopathy was documented in six patients, and five exhibited hepatomegaly and/or splenomegaly. Conjunctival injection and/or lip erythema developed in three patients during the clinical course.

**Fig 1:**
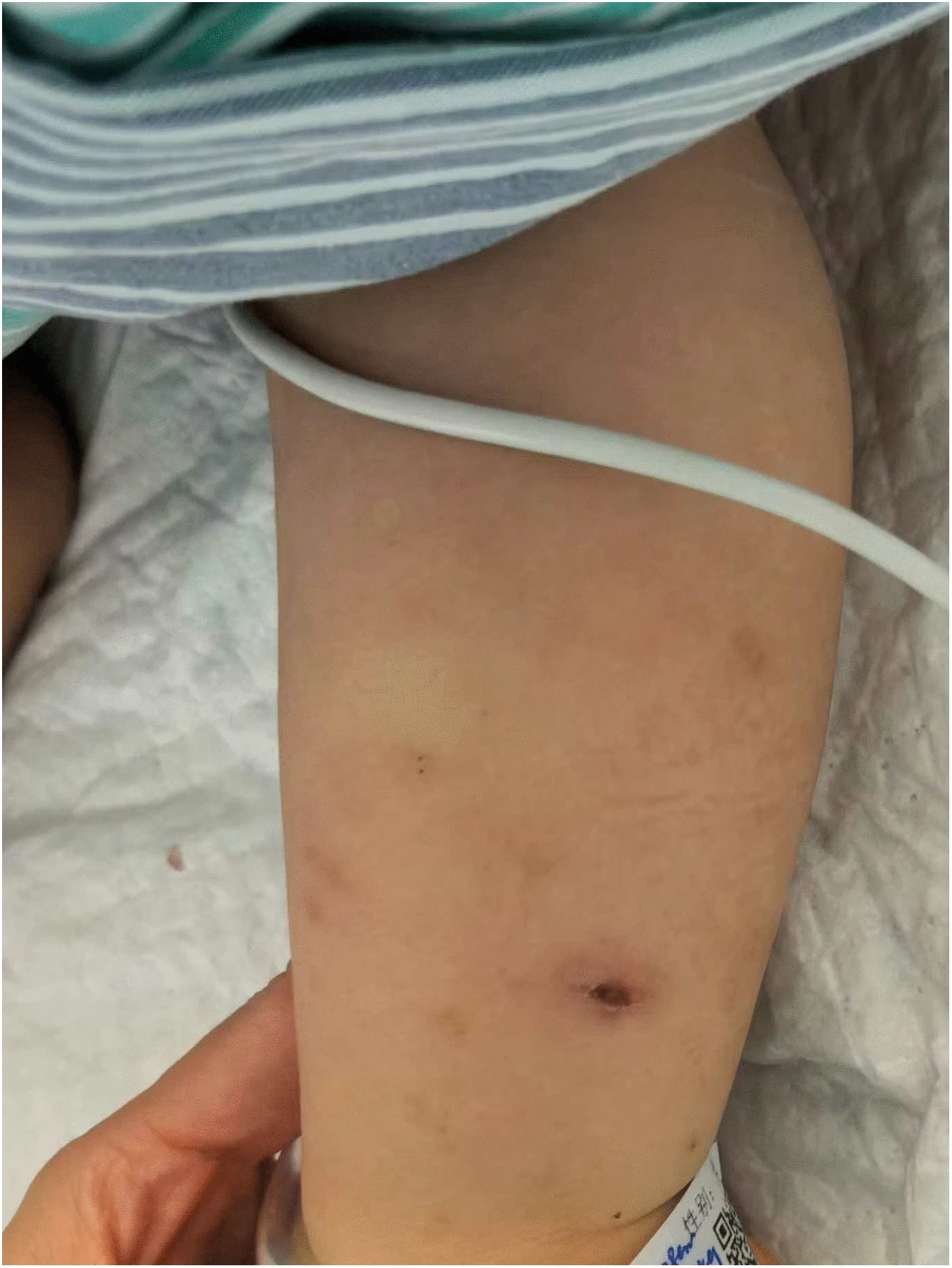
Characteristic eschar on the left lower leg of a representative patient.

### Laboratory findings

Peripheral blood leukocyte counts ranged from 7.5 to 31.4 x 10^9^/L, with neutrophil proportions of 39%-75%. Hemoglobin levels ranged from 86 to 106 g/L, and platelet counts from 63 to 384 x 10^9^/L. Atypical lymphocytes (5%-12%) were observed in all four patients who underwent peripheral blood smear examination. Alanine aminotransferase and aspartate aminotransferase were mildly to moderately elevated, and serum albumin was reduced in all patients (range: 19.7-37.0 g/L).

Serum ferritin was elevated in all six patients tested (range: 592-11,705 ng/mL). C-reactive protein (CRP) was elevated in all seven patients (range: 32-147 mg/L). Creatine kinase (CK) levels were within normal limits in all patients. N-terminal pro-B-type natriuretic peptide (NT-proBNP) was elevated in three of four patients tested (range: 2,644-6,992 pg/mL). Procalcitonin levels in six patients ranged from 0.49 to 10.3 ng/mL. Erythrocyte sedimentation rate (ESR) was elevated in three of six patients (range: 29-58 mm/h).

Immunophenotyping in three patients revealed CD4^+^ T-cell counts of 586-990 cells/mcL (15.34%-22.49%) and CD8^+^ T-cell counts of 1,572-2,562 cells/mcL (40.25%-53.8%), with uniformly decreased CD4^+^ T-cell /CD8^+^ T-cell ratios (0.30-0.50), predominantly driven by CD8^+^ T-cell expansion. B-cell counts ranged from 236 to 1,213 cells/mcL (5.12%-30.26%). Natural killer (NK) cell counts ranged from 218 to 804 cells/mcL (5.44%-20.86%); one patient had NK cell activity of 15.7%, and soluble CD25 levels were 1,226.92 and 8,539.00 U/mL in two patients.

### Pathogen detection

The Weil-Felix test (OXK) was performed in six patients between days 10 and 14 of illness; three were positive at a titer of 1:160 and three were negative. One patient underwent three sequential Weil-Felix tests, all of which were negative. Blood metagenomic next-generation sequencing (mNGS) was performed in two patients and detected O. tsutsugamushi with read counts of 355 and 7,960, respectively. One patient had both a positive Weil-Felix test and a positive blood mNGS result.

Bone marrow aspiration and cytology were performed in four patients, all of whom demonstrated hemophagocytosis.

### Imaging findings

Chest radiography in all seven patients revealed varying degrees of pulmonary infiltration and inflammation in six, with concomitant pleural effusion in three. Electrocardiography showed no significant arrhythmias in any patient. Echocardiography, performed between days 9 and 14 of illness, demonstrated the following CALs: right coronary artery (RCA) aneurysm (n = 1), left anterior descending (LAD) artery aneurysm (n = 1; Fig 2), bilateral coronary artery dilatation (n = 1), bilateral coronary artery intimal thickening (n = 3), and isolated left coronary artery (LCA) dilatation (n = 1). Detailed echocardiographic measurements and Z-scores are presented in Table 1.

**Fig 2:**
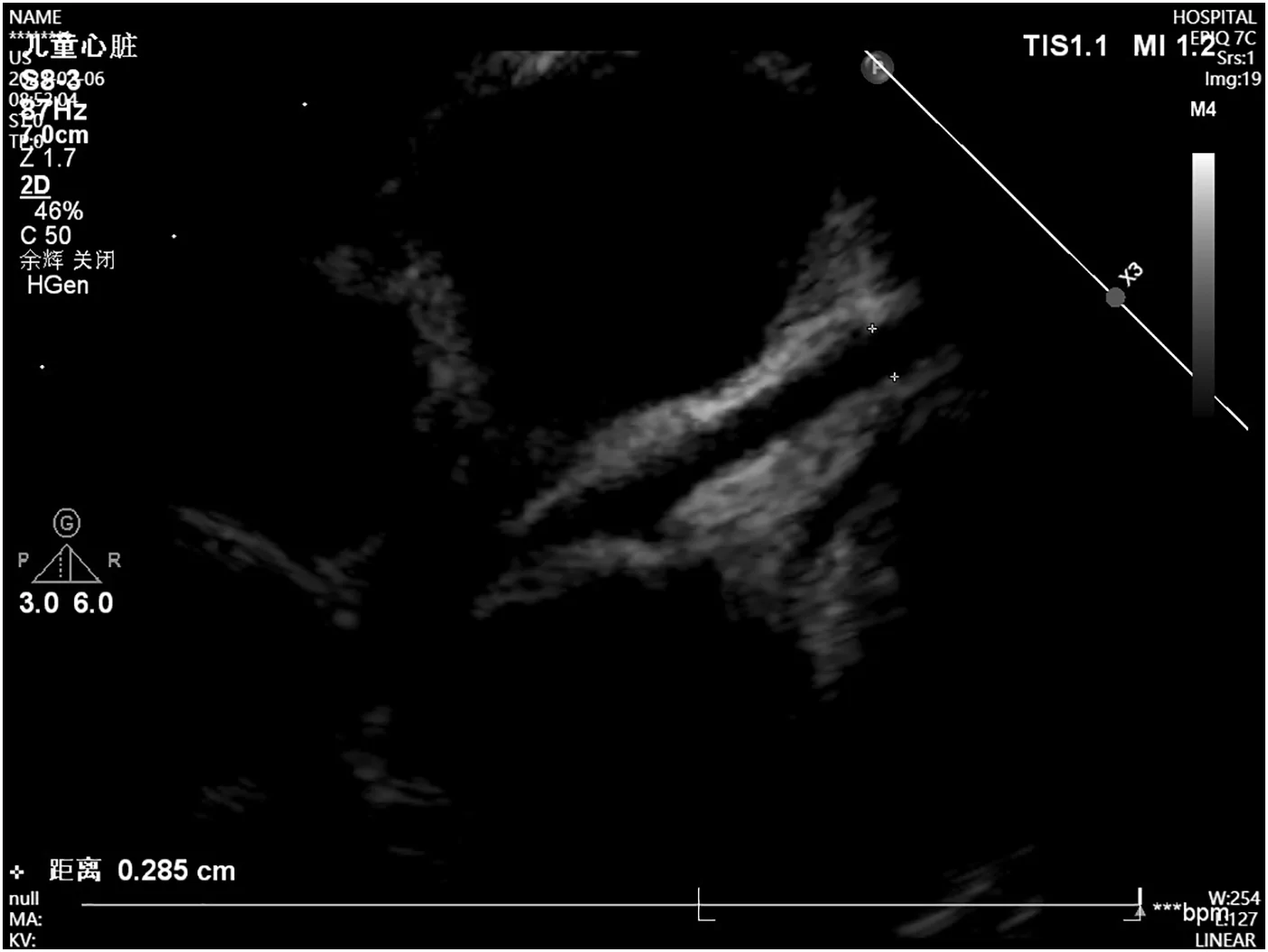
Echocardiographic findings at diagnosis for Patient 4 revealed a left anterior descending coronary artery aneurysm.

**Table 1:** Coronary artery lesion characteristics and pharmacological treatment in seven pediatric patients with scrub typhus.

Case	Age(years)	IVIGTreatment	NSAID(Type)	LMCA Diameter(mm)	LMCAZ-score	RCA Diameter(mm)	RCAZ-score	Diagnosis
1	6.0	2 g/kg	Yes (clopidogrel)	3.4	1.57	3.9	2.73	RCA aneurysm
2	8.0	2 g/kg	Yes (aspirin)	3.2	2.11	3.0	2.19	Bilateral CA dilatation
3	3.0	0.8 g/kg	No	2.9	1.50	2.3	0.84	Bilateral CA intimal thickening
4	2.0	2 g/kg (x3)	Yes (aspirin)	2.9a	2.73	2.2	0.77	LAD aneurysm
5	0.9	2 g/kg	No	2.8	1.95	2.0	0.70	Bilateral CA intimal thickening
6	11.0 mths	2 g/kg	No	2.6	2.00	N/A	N/A	LCA dilatation
7	2.0	None	No	2.8	1.64	2.4	0.90	Bilateral CA intimal thickening

IVIG, intravenous immunoglobulin; NSAID, non-steroidal anti-inflammatory drug; LMCA, left main coronary artery; RCA, right coronary artery; LAD, left anterior descending artery; LCA, left coronary artery; CA, coronary artery; N/A, not assessed.

a LAD ostial diameter: 2.5 mm; dilated segment diameter: 2.9 mm.

### Diagnosis and complications

A definitive diagnosis of scrub typhus was established in four patients, a clinical diagnosis was established in three, according to epidemiological history, typical eschar and clinical symptoms. Major complications included pneumonia (n = 5, three severe), respiratory failure (n = 3), septic shock (n = 3), hemophagocytic lymphohistiocytosis (n = 2), KD (n = 3), multiple organ dysfunction syndrome (n = 2), incomplete KD (n = 1), coagulopathy (n = 1), pulmonary hemorrhage (n = 1), and encephalopathy (n = 1).

### Treatment and Outcomes

Anti-infective therapy was initiated between days 9 and 15 of illness. Two patients (aged 6 and 8 years) received doxycycline for 7-10 days, and five patients received azithromycin (intravenous or oral) for 1-3 courses (3-9 days per course). All seven patients were initially suspected of having KD. Six of them received intravenous immunoglobulin (IVIG) accordingly, at doses of 2 g/kg (n = 5) or 0.8 g/kg (n = 1). However, all six IVIG-treated patients failed to respond, with recurrent high fever persisting for days, with or without concurrent declines in platelet counts. Notably, one patient remained febrile despite receiving two courses of IVIG at 2 g/kg. Among the six IVIG-treated patients, four met the diagnostic criteria for KD or incomplete KD. Ultimately, all seven patients—including the one who did not receive IVIG—defervesced within 1-3 days of initiating antimicrobial therapy directed at scrub typhus.

Additional supportive therapies included non-invasive and invasive mechanical ventilation, packed red blood cell transfusion, fresh frozen plasma infusion, albumin supplementation, fibrinogen replacement, and corticosteroid anti-inflammatory therapy (0.3-2 mg/kg/day). All patients received concurrent third-generation cephalosporin antibiotics during their clinical course. Hospital stay ranged from 8 to 29 days, and four patients required ICU admission (7-10 days).

Patient 1, who had an RCA aneurysm at discharge (Fig 3), continued oral clopidogrel for three months until echocardiographic normalization was achieved; repeat echocardiography three months after clopidogrel discontinuation remained normal. Patient 4, with an LAD aneurysm, continued oral aspirin post-discharge; follow-up echocardiography at two months showed residual LCA dilatation and mildly thickened RCA intima. Patients 2, 5, 6 and 7 demonstrated echocardiographic normalization of coronary arteries within 3 days to one month of anti-infective therapy. Patient 3 was lost to echocardiographic follow-up.

**Fig 3:**
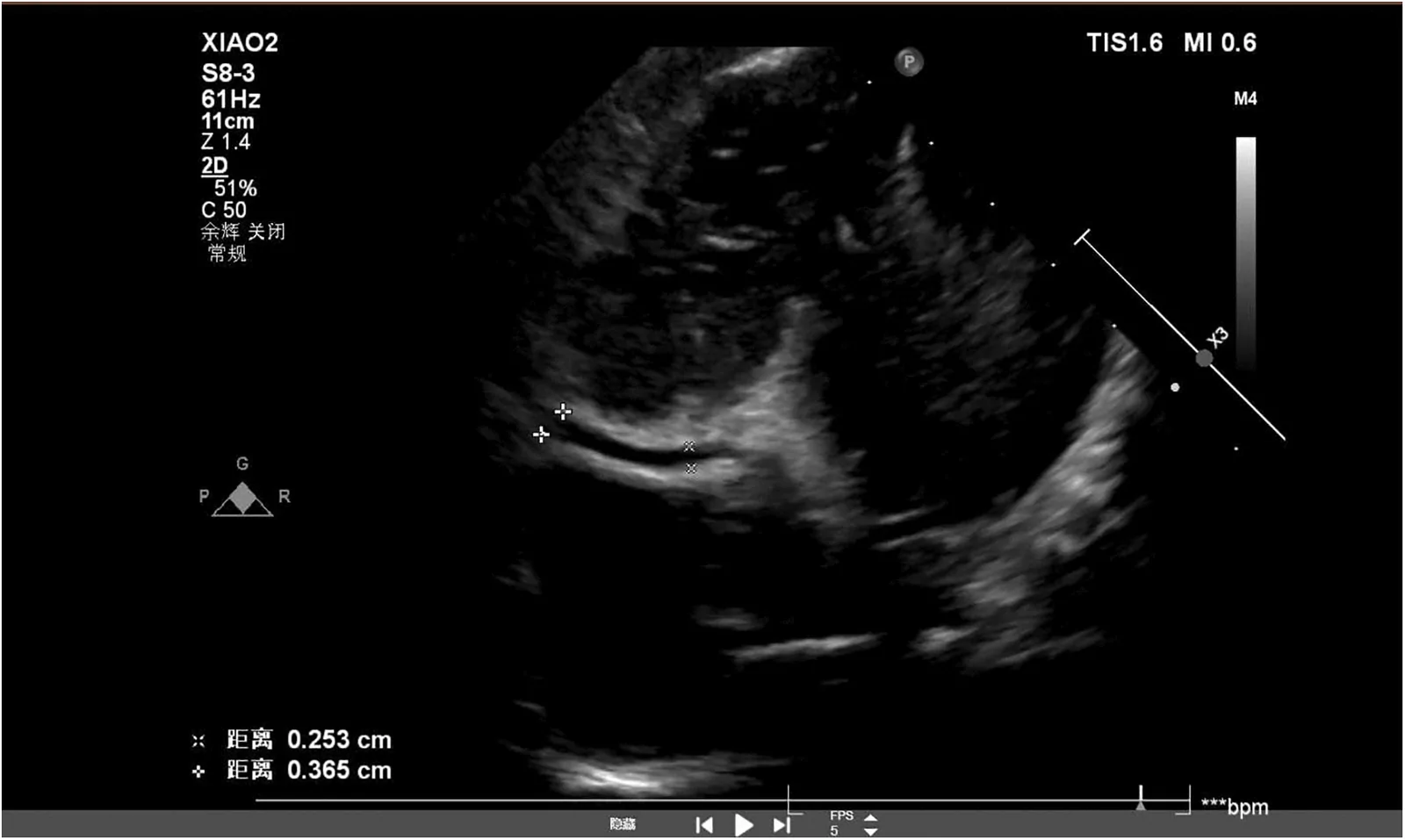
Echocardiographic findings at discharge for Patient 1 revealed a right coronary artery aneurysm.

## Discussion

This case series documents the clinical spectrum of CALs complicating pediatric scrub typhus in southern China and corroborates the emerging recognition that O. tsutsugamushi infection can produce coronary artery pathology resembling, and occasionally coinciding with, KD. To our knowledge, this represents the first systematic characterization of scrub typhus-associated CALs in a pediatric cohort in China.

The epidemiological profile of our cohort aligns with known regional patterns. The incidence of scrub typhus in Guangzhou has risen steadily from 2006 to 2019, with positive correlations observed between disease incidence and mean temperature, cumulative rainfall, relative humidity, sunshine duration, population density, and green space coverage [11]. As a subtropical city with abundant vegetation, Guangzhou warrants heightened attention for scrub typhus prevention and control. The female predominance in our series (5/7, 71%) is consistent with prior reports of higher scrub typhus incidence among women [12]. The seasonal distribution — predominantly May through September with a June peak — corresponds to the reported scrub typhus epidemic season of April through September in China [13].

The intersection of scrub typhus and KD presents a clinically important diagnostic dilemma. Scrub typhus misdiagnosed as KD is not infrequent [6], while concurrent scrub typhus and KD appears to be rare [7]. In 2020, Hwang et al. reported the first two cases of KD with positive O. tsutsugamushi serology; both patients exhibited typical KD features — fever persisting beyond five days, conjunctival injection, lip erythema, cervical lymphadenopathy, and coronary artery dilatation — and both improved rapidly with macrolide antibiotics, IVIG, and oral aspirin, with normalized coronary arteries at eight-week follow-up [7]. Those findings suggested a possible etiological link between O. tsutsugamushi infection and KD onset, and that KD may occur at or shortly after the onset of scrub typhus.

The immunopathogenesis of infection-triggered KD has been increasingly elucidated. KD is understood as a pediatric vasculitis syndrome whose etiology and pathogenesis remain incompletely defined; however, converging evidence implicates immune dysregulation provoked by infectious agents through several mechanisms: (i) direct viral infection; (ii) bacterial superantigen-mediated immune activation; (iii) innate immune pathway engagement via pathogen-associated molecular patterns and complement activation; and (iv) host genetic susceptibility modulating the response to infectious stimuli [5]. The association between KD-like presentations and diverse infections further supports infection-driven immunosuppression or dysregulation as a core pathogenic process [14,15]. Two paradigmatic conditions illustrate this connection: HIV-associated Kawasaki-like syndrome (KLS) and Multisystem Inflammatory Syndrome in Children (MIS-C) [14,16,17]. HIV-KLS typically emerges in adults with low CD4 counts and high viral loads, displaying inflammatory profiles akin to those of pediatric KD [14,15]. In contrast, MIS-C follows SARS-CoV-2 infection and is characterized by transient severe lymphopenia, which precipitates immune dysregulation and hyperinflammation [16,17]. Although both syndromes involve immune disturbances, MIS-C more frequently presents with myocardial injury, whereas coronary artery lesions remain more characteristic of KD [16,17]. Collectively, these examples demonstrate that either chronic (HIV) or transient (COVID-19) immunosuppression can precipitate KD-like vasculitis, reinforcing the clinical overlap observed in our scrub typhus cases [14–17].

Supporting this overlap, Mahajan et al. (2022) reported that among 10 Indian children aged 1 month to 11 years diagnosed with KD during treatment for various infections — including dengue, chikungunya, SARS-CoV-2, hepatitis A, tuberculosis, brucellosis, disseminated staphylococcal sepsis, scrub typhus, and enteric fever — eight had echocardiographic evidence of coronary artery involvement, including one patient with concurrent scrub typhus and KD [18]. Earlier work by Pesonen et al. demonstrated that infectious diseases can drive coronary artery intimal thickening in children through postmortem histopathological analysis [10,19]. Furthermore, a 2014 nationwide cohort study from Taiwan found that scrub typhus patients over 20 years of age had a 37% increased risk of developing acute coronary syndrome within one year of infection [20], underscoring the long-term cardiovascular implications of O. tsutsugamushi infection whose pathophysiological mechanisms warrant further investigation.

The CALs observed in our series — coronary artery aneurysm, dilatation, and intimal thickening — mirror the spectrum of coronary pathology seen in KD. The marked elevation of inflammatory markers, particularly ferritin and CRP, indicates a robust hyperinflammatory state. Furthermore, the cardiovascular manifestations and coagulopathy observed in our patients suggest widespread endothelial damage leading to systemic microvascular dysfunction, as other study showed [21],representing an additional pathophysiological mechanism contributing to coronary artery injury. The expansion of CD8 + T cells with a concomitant decrease in the CD4 + T-cell/CD8 + T-cell ratio suggests that immune dysregulation, characterized by a predominant cytotoxic T-cell response, may contribute to disease severity and the development of coronary complications. The identification of hemophagocytosis on bone marrow examination in four patients further supports a profound immune activation syndrome. Indeed, scrub typhus complicated by hemophagocytic lymphohistiocytosis has been previously reported and is readily misdiagnosed; anti-infective therapy alone can ameliorate both the clinical and laboratory abnormalities of the hemophagocytic syndrome [22,23].

From a therapeutic standpoint, our findings carry important implications. Meta-analytic evidence indicates that azithromycin is non-inferior to doxycycline for pediatric scrub typhus [24], and doxycycline use in children under eight years has not been associated with demonstrable dental enamel hypoplasia [25]. In our cohort, both azithromycin and doxycycline achieved defervescence within 1-3 days, consistent with excellent clinical efficacy. Early initiation of macrolide antibiotics is critical for improving outcomes in pediatric scrub typhus; however, the Weil-Felix test — the most commonly used diagnostic assay at our institution — has a reported sensitivity of only 15.82% [26], substantially limiting its utility for early diagnosis. The pathognomonic eschar remains a vital diagnostic clue, with a reported specificity approaching 100% [27]. In our series, three patients underwent two or more Weil-Felix tests, all of which were negative; the diagnosis was ultimately established based on the presence of typical eschars, epidemiological history, and a favorable response to empirical doxycycline or azithromycin therapy.

While eschar is a highly specific clue [28], its absence itself is a risk factor for severe disease [28,29]. The diagnostic value of mNGS in challenging scrub typhus cases deserves particular emphasis. Recent reports have demonstrated that mNGS can successfully detect O. tsutsugamushi in patients presenting with atypical manifestations such as optic neuritis, refractory septic shock, and multi-organ dysfunction, establishing this technology as an effective tool for early diagnosis [30–32]. In our cohort, all patients were diagnosed more than eight days after symptom onset, and 57% (4/7) developed severe complications including respiratory failure and severe pneumonia. Given that delayed diagnosis (beyond day 8 of illness) is associated with increased mortality [33], and considering the low sensitivity of the Weil-Felix test, early mNGS testing should be considered for patients with a high clinical suspicion of scrub typhus who lack a typical eschar, as this approach may facilitate timely diagnosis, reduce severe complications, and improve outcomes.

A particularly instructive observation was that the four patients with concurrent KD or incomplete KD who failed to defervesce after adequate IVIG (2 g/kg) all responded promptly — within 1-3 days — to macrolide antibiotics. This observation corroborates that identifying the underlying etiology is paramount in the management of scrub typhus-associated CALs and suggests that IVIG alone is insufficient to control fever when O. tsutsugamushi infection is the primary driver. The detailed mechanisms underlying this phenomenon require further investigation. Additionally, the genetic diversity of O. tsutsugamushi serotypes is considered a potential contributor to variations in disease incidence; highly homologous Karp genotype strains have been detected in the Guangzhou region [34]. Although our sample size precluded genotype analysis, future studies should explore the relationship between bacterial genotype and clinical phenotype.

This study has several limitations. The small sample size, inherent to a single-center case series, limits the generalizability of our findings. The retrospective design precluded standardized echocardiographic follow-up intervals and uniform laboratory testing. One patient was lost to echocardiographic follow-up, and the long-term cardiovascular outcomes of scrub typhus-associated CALs remain unknown. Furthermore, the Weil-Felix test was the primary serological diagnostic tool; more sensitive methods such as indirect immunofluorescence assay were not available.

In conclusion, O. tsutsugamushi infection can cause a spectrum of coronary artery pathology including aneurysm, dilatation, and intimal thickening in children. For patients with suspected KD or incomplete KD who respond poorly to IVIG, clinicians should pursue a thorough history of insect bites and environmental exposures, meticulously examine for the pathognomonic eschar or ulcer, and promptly perform Weil-Felix testing and mNGS where available. Early etiological diagnosis and initiation of macrolide antibiotics are associated with favorable outcomes in children with scrub typhus complicated by CALs.

## Supporting information

S1 Table Basic clinical data of the seven patients. (XLSX)
